# Consistent Provisions Mitigate Exposure to Sexual Risk and HIV Among Young Adolescents in South Africa

**DOI:** 10.1007/s10461-019-02735-x

**Published:** 2019-11-20

**Authors:** Elona Toska, Laurence Campeau, Lucie Cluver, F. Mark Orkin, McKenzie N. Berezin, Lorraine Sherr, Christina A. Laurenzi, Gretchen Bachman

**Affiliations:** 1 AIDS and Society Research Unit, University of Cape Town, Cape Town, South Africa; 2 Department of Sociology, University of Cape Town, Cape Town, South Africa; 3 Department of Social Policy and Intervention, University of Oxford, Oxford, UK; 4 Department of Child and Adolescent Psychiatry, Cape Town, South Africa; 5 SAMRC Developmental Pathways for Health Research Unit, School of Clinical Medicine, University of Witwatersrand, Johannesburg, South Africa; 6 Department of Applied Psychology, New York University, New York, NY USA; 7 Institute of Global Health, University College London, London, UK; 8 Institute for Life Course Health Research, Faculty of Medicine and Health Sciences, Stellenbosch University, Tygerberg, South Africa; 9 Office of HIV/AIDS, United States Agency for International Development, Arlington, VA USA; 10 University of Cape Town, 4.89 Leslie Social Science Building, 12 University Avenue South, Rondebosch, 7700 Cape Town, South Africa

**Keywords:** Adolescents, South Africa, Sexual risk, HIV, Prevention, Adolescentes, Sudáfrica, Riesgo sexual, VIH, Prevención

## Abstract

Exposure to sexual risk in early adolescence strongly predicts HIV infection, yet evidence for prevention in young adolescents is limited. We pooled data from two longitudinal South African surveys, with adolescents unexposed to sexual risk at baseline (n = 3662). Multivariable logistic regression tested associations between intermittent/consistent access to eight provisions and reduced sexual risk exposure. Participants were on average 12.8 years, 56% female at baseline. Between baseline and follow-up, 8.6% reported sexual risk exposure. Consistent access to caregiver supervision (OR 0.53 95%CI 0.35–0.80 p = 0.002), abuse-free homes (OR 0.55 95%CI 0.37–0.81 p = 0.002), school feeding (OR 0.55 95%CI 0.35–0.88 p = 0.012), and HIV prevention knowledge (OR 0.43, 95%CI 0.21–0.88 p = 0.021) was strongly associated with preventing early sexual risk exposure. While individual factors reduced the odds of sexual risk exposure, a combination of all four resulted in a greater reduction, from 12.9% (95%CI 7.2–18.7) to 1.0% (95%CI 0.2–1.8). Consistent access to provisions in early adolescence may prevent sexual risk exposure among younger adolescents.

**Electronic supplementary material:**

The online version of this article (10.1007/s10461-019-02735-x) contains supplementary material, which is available to authorized users.

## Resumen

La exposición al riesgo sexual en la adolescencia temprana predice fuertemente la infección por VIH, pero la evidencia para la prevención en adolescentes jóvenes es limitada. Reunimos datos de dos encuestas longitudinales sudafricanas, con adolescentes no expuestos al riesgo sexual al inicio del estudio (n = 3662). La regresión logística multivariable probó las asociaciones entre el acceso intermitente/constante a ocho disposiciones potenciales y la exposición al riesgo sexual reducida. Los participantes fueron en promedio 12.8 años, 56% mujeres. Entre el inicio y el seguimiento, el 8.6% informó exposición al riesgo sexual. Acceso constante a supervisión de un cuidador (OR 0.53 95%CI 0.35–0.80 p = 0.002), las casas sin abuso (OR 0.55 95%CI 0.37–0.81 p = 0.002), alimentación escolar (OR 0.55 95%CI 0.35–0.88 p = 0.012), y conocimiento de prevención del VIH (OR 0.43, 95%CI 0.21–0.88 p = 0.021) estuvo fuertemente asociado con la prevención de la exposición temprana al riesgo sexual. Si bien los factores individuales redujeron las probabilidades de exposición al riesgo sexual, una combinación de los cuatro resultó en una mayor reducción, de 12.9% (IC 95% 7.2–18.7) a 1.0% (IC 95% 0.2–1.8). El acceso constante a las disposiciones en la adolescencia temprana puede prevenir la exposición al riesgo sexual entre los adolescentes más jóvenes.

## Introduction

Despite overall reductions in total HIV incidence in Eastern and Southern Africa, rates of new infections among 15- to 24-year-old adolescent girls and young women remain unacceptably high, with an estimated 7000 new infections each week [1, 2]. The risk of HIV infection increases progressively with age, with a significant increase among 16- to 17-year-old adolescent girls and 20- to 24-year-old young men [3]. This persistent incidence coincides with a rapidly growing adolescent population in the region, from 229 million in 2015 to an estimated 435 million in 2030 [4, 5]. Preventing new HIV infections among adolescents in Eastern and Southern Africa is critical in order to reduce adolescent mortality and morbidity [6].

Evidence examining exposure to sexual risk has largely focused on 15- to 24-year-old adolescent girls and young women, particularly given the strong association between sexual risk exposure and heterosexual HIV infection in this population in sub-Saharan Africa [7–9]. Early sexual debut, sex in exchange for goods or money, and age-disparate relationships among young women and older sexual partners also contribute to high HIV infection risk [10–16], including unintended pregnancies [17].

Evidence suggests that multi-level factors affect early exposure to sexual risk. Specifically, individual-level factors (poor knowledge of HIV transmission and prevention, low self-efficacy, and violence victimization), family-based factors (orphanhood, parent/caregiver-adolescent engagement and parental monitoring), household-based factors (poverty and food insecurity) [18], and school dropout [19–22] are closely associated with early exposure to sexual risk, including HIV infection. Supporting adolescents to remain in school protects them from HIV infection, unintended adolescent pregnancy, and sexually transmitted infections [23], especially for adolescent girls [24, 25]. To prevent new HIV infections among adolescents and young people, interventions need to address risk factors across the socio-ecological continuum [26–29]. However, among adolescents who remain enrolled in school, data is still needed to indicate which specific school-based provisions are most effective at preventing HIV infections and reducing early exposure to sexual risk. In particular, evidence on which types of subsidies should be applied to which subgroups of adolescents would support more effective delivery of HIV and sexual risk prevention services.

While we know about risk and protective factors for HIV infection among older adolescent girls and young women, there is less evidence on sexual risk among adolescents living with HIV [29]. Research on reducing sexual risk exposure among young adolescents, for those who are HIV uninfected as well as those living with HIV, is critical to breaking the cycle of HIV transmission. Reducing exposure to sexual risk has been associated with reduced new infections among young people and adults [30, 31]. For instance, increasing condom use amongst adolescents is a key strategy found to effectively reduce exposure to HIV among adolescents and young people [32]. Despite this data, evidence is needed to know what works in early adolescence, when many of these sexual risks first emerge, including which combinations of provisions have the highest impact to prevent exposure to sexual risk [33].

The evidence on HIV prevention efforts for adolescents and young people highlights the need to combine biomedical, individual and structural interventions to address the causal pathways to HIV infection during this vulnerable age [33–35]. Programming for younger adolescents is critical to developing resilience, initiating safe behaviors, and enabling early detection of risk factors [36]. However, improved access to quality and consistent services and programming for adolescents and young people is needed to achieve the highest reduction in HIV incidence [37]. Findings from a large dataset of South African adolescents showed that consistent access to the government-issued Child Support Grant was associated with reduced HIV risk behaviors [38]. Additional evidence on the effect of the timing and consistency of accessing HIV prevention programming for adolescents is needed, beyond government cash transfers. Longitudinal analyses are needed to show if, and how, access to single or combinations of services or provisions shapes early exposure to sexual risk, particularly among an age group rarely included in such research: 10- to 14-year-old adolescents.

We use data from two longitudinal data sets from two South African surveys to answer three research questions: (1) which provisions (alone and in combination) are associated with preventing exposure to sexual risk in early adolescence, (2) do associations of provisions with reduced sexual risk exposure vary by gender and residential location (urban/rural), and (3) how do differing levels of access to provisions over time influence early exposure to sexual risk?

## Methods

This analysis draws upon the pooling of individual-level data from two existing independent longitudinal cohort studies, Young Carers and Mzantsi Wakho, based in three provinces of South Africa. The Young Carers study is a prospective cohort of n = 3401 adolescents aged 10–17 living in two South African provinces, Mpumalanga and Western Cape. The cohort collected data between 2010 and 2012. Participants were recruited through door-to-door recruitment of two randomly-selected census enumeration areas in a rural and urban district in each province [39]. The Mzantsi Wakho study is a prospective cohort of n = 1410 10- to 19-year-old adolescents of sero-assorted HIV status living in a health sub-district in the Eastern Cape province in South Africa, with data collected from 2014 to 2017. The longitudinal Mzantsi Wakho sample includes 1080 adolescents living with HIV, an important population for secondary HIV prevention efforts. The studies shared investigators and used similar data collection procedures, which are explained in more detail in prior publications [39, 40]. The studies had high acceptability with low refusal rates (< 4% at both baseline and follow-up). During follow-up, 96.7% of Young Carers and 93.8% of Mzantsi Wakho participants were re-interviewed. Follow-up times averaged 353 days for Young Carers (range 77–829 days) and 571 days for Mzantsi Wakho (range 210–1091 days). Ethical approvals for data collection were obtained from Universities of Oxford (SSD/2/3/IDREC and SSD/CUREC2/12-21), Cape Town (HREC 389/2009 and CSSR 2013/04), and the relevant provincial South African Departments of Health, Basic Education, and Social Development [39, 41].

### Data Pooling

To minimize measurement bias, we pooled data following a rigorous examination of study data collection tools for conceptual and measurement validity [42]. All items were measured using identical tools across the two surveys, except for HIV knowledge, as detailed below. Questions measured the same phenomena within comparable time frames, and answer options were comparable for categorical variables. In cases where variables had a different number of categories between studies, the variable with the larger number of categories was reduced to be equivalent to the variable with the least categories [43]. We conducted a quality control check following variable harmonization, using cross-tabulations for categorical variables and five-number summary for continuous variables by sample. We investigated dissimilar category counts to ensure that differences in frequencies reflected actual differences between population, and not difference in definitions or data collection procedures. After completing this process, we computed estimates on the resulting dataset as one larger sample, controlling for original study location in each analysis.

### Ethics and Data Collection Procedures

All participants 18 and over provided voluntary informed consent, and both caregiver and adolescent provided assent/consent if participants were under 18 years old. Researchers fluent in local languages (Xhosa, Swati, Tsonga, and Sotho), who were trained to discuss sensitive topics with children and adolescents, conducted face-to-face interviews. Questionnaires were co-designed in consultation with a teen advisory group and piloted with adolescents from the study areas. Participants were interviewed in a location of their choosing, which maximized safety, confidentiality, and comfort throughout the interview. No financial remuneration was awarded for participating, but all adolescents received a snack pack and a study participation certificate. Participants at risk of harm or experiencing harm at the time of interview received support to link with emergency or support services, based on the recommendations of a trained social worker.

### Measures

Full questionnaires are available at www.mzantsiwakho.co.za and www.youngcarers.org.za. The primary *study outcome* was ***exposure to sexual risk***, measured as the incidence of at least one of four high-risk sexual experiences between baseline and follow-up: inconsistent condom use, transactional sex, age-disparate relationship, or early sexual debut. *Inconsistent condom use* referred to ‘never, ‘rarely’ or ‘sometimes’ using condom during sexual intercourse in the past year. *Transactional sex* was past-year incidence of sex in exchange for food, shelter, school fees, transport, or money. *Age*-*disparate relationship* was past-year incidence of having a sexual partner more than 5 years older. *Early sexual debut* referred to having had a first sexual intercourse before the age of 18 years old. *Past*-*year pregnancy/having made someone pregnant* was recorded through self-reports for both adolescent girls and boys.

### Provisions

A total of eight provisions were hypothesized to delay sexual risk exposure based on the existing evidence base: (1) strong parental/caregiver supervision; (2) positive parenting/caregiving; (3) abuse-free homes; (4) school feeding; (5) affordable school materials; (6) affordable school fees; (7) government cash transfers; and (8) HIV prevention knowledge. Adolescents were recorded as experiencing or having access to these provisions or not at baseline and follow-up, and access was categorized as (1) no access at either baseline or follow-up; (2) intermittent access, defined as access at baseline *or* follow-up; (3) consistent access, measured as access at both baseline and follow-up.

*Parenting/caregiver support* was measured using two sub-scales of the Alabama Parenting Questionnaire [44, 45]. *Strong parental/caregiver supervision* was computed as adolescents choosing ‘very good’ on all six items about home rule-setting or monitoring of adolescent socializing, while positive parenting was measured as scoring ‘very good’ on all five items about caregiver-provided praise and positive reinforcement. Living in an *abuse*-*free home* was measured through adolescent self-reported monthly or more frequent experiences of physical or emotional violence at home [46]. Adolescent access to three types of educational subsidies was measured through adolescent self-reported access to: *school feeding*, defined as accessing at least one meal a day at school; *affordable uniforms and school materials*, measured as the adolescent’s family being able to afford uniforms and school stationery; and *affordable school fees*, defined as access to fee-free schooling. Each subsidy was included as a separate provision in analyses. *Government cash transfers* was defined as access to at least one child-related government grant into the home, such as South Africa’s Child Support Grant or Foster Child Grant. *HIV prevention knowledge* was measured as adolescent’s correct knowledge of four items on different modes of HIV transmission and prevention methods, for example ‘HIV cannot be passed from a HIV-positive mother to her unborn child.’

Additionally, analyses controlled for eight covariates across analyses, pre-selected for their potential effects on sexual risk exposure or access to social protection: (1) age; (2) gender; (3) urban/rural location; (4) formal/informal housing type; (5) province of residence; (6) socio-economic status (households were defined as poor if the adolescent reported missing at least one of eight basic necessities, such as “money to go to the doctor”, or “three meals a day”); (7) orphanhood, defined as being both maternally and paternally orphaned, and (8) HIV status, determined through self-reported HIV-positive test or being on antiretroviral therapy.

### Analyses

The final pooled dataset consisted of n = 4811 participants. All participants who had been exposed to sexual risk prior to baseline data collection were excluded from analysis (n = 1149), to allow the analyses to identify provisions associated with exposure to sexual risk. Of the n = 3662 (76%) participants who reported no exposure to sexual risk at baseline, 97% were enrolled in school at both baseline and follow-up. We focused our analyses among adolescents enrolled in publicly-funded government schools in both data points, given high rates of enrolment at both times in this young sample, which reflect enrolment rates in other studies in South Africa [47]. To explore which provisions were associated with the outcome, we conducted final analyses using Stata 15 among n = 3635 adolescents who did not report sexual risk at baseline and were enrolled at school at both times, with exposure to sexual risk at follow-up as the outcome. Missing data was minimal (< 1%) for all included variables.

First, we calculated descriptive statistics for all covariates, provisions included. We used comparison tests to compare adolescents exposed to sexual risk at follow-up and those who were not exposed, using two-sample t-tests for continuous variables and Pearson’s χ^2^ tests for categorical variables (Table 1). We reported frequency distributions for longitudinal access to provisions (Table 2).

**Table 1:** Socio-demographic characteristics

Variables at baseline^a^	Total sample (n, %)	No sexual risk (n, %)	Early sexual risk exposure (n, %)	Comparison test (p-value)^b^
Age (mean, range)	12.8 (9–19)	12.7 (9–19)	14.4 (10–19)	< 0.0001
9	10, 0.3	10, 0.3	0, 0	
10	555, 15.3	545, 16.4	10, 3.2	
11	514, 14.1	498 15.0	16, 5.1	
12	628, 17.3	604, 18.2	24, 7.6	
13	602, 16.6	562, 16.9	40, 12.7	
14	537, 14.8	469, 14.1	68, 21.7	
15	387, 10.7	311, 9.4	76, 24.2	
16	229, 6.3	185, 5.6	44, 14.0	
17	133, 3.7	108, 3.3	25, 8.0	
18	28. 0.8	21, 0.6	7, 2.2	
19	12, 0.3	8, 0.2	4, 1.3	
Age at follow-up (mean, range)	14.1 (10-21)	14.0 (10-21)	16.0 (11-21)	< 0.0001
Gender	–	–	–	0.897
Female	2048, 56.3	1870, 56.3	178, 56.7	–
Male	1587, 43.7	1451, 43.7	136, 43.3	–
Location type	–	–	–	< 0.0001
Urban	2110, 58.0	1886, 56.8	224, 71.3	–
Rural	1524, 42.0	1434, 43.2	90, 28.7	–
Housing type	–	–	–	0.349
Formal	2733, 75.2	2490, 75.0	243, 77.4	–
Informal	901, 24.8	830, 25.0	71, 22.6	–
Province of residence	–	–	–	< 0.0001
Western Cape	1154, 31.8	1087, 32.7	67, 21.3	–
Mpumalanga	1412, 38.8	1363, 41.0	49, 15.6	–
Eastern cape	1069, 29.4	871, 26.2	198, 63.1	–
Socio-economic status	–	–	–	0.12
Any missing necessities at home	2659, 73.3	2442, 73.6	217, 69.6	–
No missing necessities at home	969, 26.7	874, 26.4	95, 30.5	–
Double orphanhood	–	–	–	0.004
No	3393, 93.3	3112, 93.7	281, 89.5	–
Yes	242, 6.7	209, 6.3	33, 10.5	–
HIV status (follow-up)	–	–	–	< 0.0001
HIV-positive	835, 23.2	694, 21.1	141, 45.2	–
HIV-negative	2771, 76.8	2600, 78.9	171, 54.8	–

^a^All variables reported in this table were measured at baseline unless noted otherwise

^b^Two-sample t-tests were used for continuous variables and Pearson’s χ^2^ tests were used for categorical variables

**Table 2:** Access to supportive and protective provisions

Provisions	No access (n, %)	Intermittent access (n, %)	Consistent access (n, %)
Strong parental/caregiver supervision	1088, 30.0	1598, 44.1	940, 25.9
Positive parenting	1601, 44.0	1388, 38.2	646, 17.8
Abuse-free home	856, 23.6	1553, 42.8	1224, 33.7
School feeding scheme	282, 7.8	310, 8.6	3033, 83.7
Affordable school materials	2043, 56.2	1021, 28.1	571, 15.7
Affordable school fees	189, 5.2	797, 21.9	2649, 72.9
Government cash transfers	284, 7.9	626, 17.3	2706, 74.8
HIV prevention knowledge	2818, 86.0	390, 11.9	69, 2.1

Second, to assess the validity of the self-reported study outcome, we tested whether exposure to sexual risk was associated with incident self-reported pregnancy or having made someone pregnant, using Pearson’s χ^2^ test.

Third, we assessed associations between each level of access to provisions and exposure to sexual risk in multivariable logistic regressions using the three-step Hosmer-Lemeshow approach [48] (Table 3). In Model 1, we included all factors in a multivariable logistic regression with exposure to sexual risk as the outcome, controlling for all covariates. In Model 2, only variables associated with reduced sexual risk exposure at p < 0.1 in the previous model were included. In Model 3, we only included variables associated with the outcome at p < 0.05 level in the previous model.

**Table 3:** Multivariable regression model of provisions associated with incident sexual risk exposure

Outcome measure: incident high-risk sex	Model 1			Model 2			Model 3		
	Odds ratio (OR)	p-value	95% CI	OR	p-value	95% CI	OR	p-value	95% CI
Age	1.43	< 0.0001	1.33–1.54	1.42	< 0.0001	1.32–1.52	1.42	< 0.0001	1.32–1.52
Gender—female	1.01	0.994	0.77–1.33	–	–	–	–	–	–
Rural residence	0.82	0.222	0.60–1.13	–	–	–	–	–	–
Informal housing (reference category: formal)	1.04	0.835	0.73–1.49	–	–	–	–	–	–
Province (reference category: Eastern Cape)									
Western Cape	0.20	< 0.0001	0.11–0.35	0.19	<0.0001	0.13–0.27	0.17	<0.0001	0.12–0.25
Mpumalanga	0.09	< 0.0001	0.05–0.16	0.08	<0.0001	0.05–0.12	0.07	<0.0001	0.05–0.11
Socio-economic status (reference category: household poverty*)*	0.87	0.499	0.58–1.31	–	–	–	–	–	–
Double orphanhood	0.86	0.518	0.53–1.37	–	–	–	–	–	–
HIV status (positive)	1.04	0.876	0.67–1.60	–	–	–	–	–	–
Supportive factors or provisions									
Parental/caregiver support—strong parental supervision^a^									
Intermittent access	0.76	0.082	0.55–1.04	0.75	0.072	0.55–1.03	0.76	0.082	0.56–1.04
Consistent access	0.52	0.002	0.34–0.78	0.53	0.002	0.35–0.80	0.53	0.002	0.35–0.80
Parental/caregiver support – Positive parenting									
Intermittent access	0.78	0.155	0.56–1.10	–	–	–	–	–	–
Consistent access	0.99	0.958	0.66–1.48	–	–	–	–	–	–
Abuse-free homes									
Intermittent access	0.96	0.804	0.67–1.36	0.92	0.636	0.65–1.30	0.92	0.648	0.66–1.30
Consistent access	0.57	0.006	0.39–0.85	0.54	0.002	0.37–0.79	0.55	0.002	0.37–0.81
School feeding scheme									
Intermittent access	0.88	0.687	0.48–1.62	0.85	0.585	0.46–1.54	0.86	0.615	0.47–1.55
Consistent access	0.61	0.054	0.36–1.01	0.57	0.025	0.35–0.93	0.55	0.012	0.35–0.88
Educational subsidies—affordable school materials									
Intermittent access	1.52	0.03	1.04–2.22	1.34	0.059	0.99–1.82	–	–	–
Consistent access	1.15	0.654	0.62–2.14	1.02	0.942	0.63–1.64	–	–	–
Educational subsidies – Fee-free school or affordable fees									
Intermittent access	0.78	0.402	0.43–1.40	–	–	–	–	–	–
Consistent access	0.78	0.375	0.44–1.36	–	–	–	–	–	–
Government cash transfers									
Intermittent access	1.08	0.799	0.58–2.01	–	–	–	–	–	–
Consistent access	1.20	0.531	0.68–2.12	–	–	–	–	–	–
HIV prevention knowledge									
Intermittent access	0.71	0.073	0.49–1.03	0.69	0.045	0.47–0.99	0.68	0.044	0.47–0.99
Consistent access	0.43	0.022	0.21–0.88	0.43	0.023	0.21–0.89	0.43	0.021	0.21–0.88

^a^All results for provisions accessed intermittently or consistently use no access as a reference category

Fourth, we tested potential interactions between all significant provisions to test for multiplicative effect. We then tested whether gender or rural residence moderated the association between the provisions and early sexual risk exposure, in light of evidence suggesting that HIV prevention programming may have different results by gender [49] and location of delivery. We tested moderation using two-way interaction terms of each factor and either gender or rural residence in multivariable regressions, including all other covariates.

**Fig. 1:**
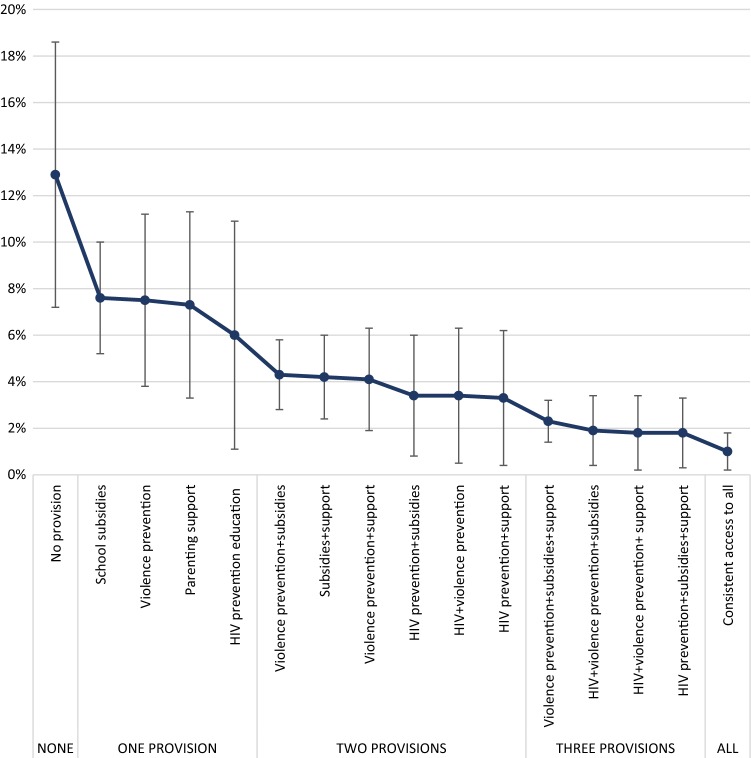
Probability of incident sexual risk exposure by combination of consistent access to provisions

Lastly, we entered all provisions significantly associated with the outcome in the final model into a marginal effect model using multivariable logistic regression with covariates held at their mean values (Table 3). We calculated predicted probabilities of the outcome according to level of access to provisions (no access, intermittent access, consistent access) as well as according to the type of access (no intervention, single intervention or combination of all interventions) (Fig. 1).

## Results

### Participant Characteristics and Outcome

Participants were, on average, 12.8 years old (SD 2.0, range 9–19 years) at baseline and 14.1 years old at follow-up (SD 2.1, range 10–21 years), and 56% were female. They lived in three provinces (Eastern Cape 29.4%, Western Cape 31.8% and Mpumalanga 38.8%), with over half living in urban areas (58%), the majority of whom were participants from urban communities in the Eastern Cape. Three-quarters lived in formal housing; another three-quarters lived in households lacking at least one basic necessity, such as food or sufficient money for school fees. Overall, 6.7% of the sample were orphaned.

Notably, 8.6% of participants reported sexual risk exposure between baseline and follow-up. This self-reported outcome was strongly associated with reporting pregnancy or having made someone pregnant during the study period incident pregnancy (Pearson’s χ^2^ (1) = 109.5, p ≤ 0.001). In bivariate analyses, adolescents who reported exposure to sexual risk were older, more likely to be orphaned, living in urban areas, and living in the Eastern Cape (Table 1).

### Access to Provisions

Three levels of access to a total of eight provisions are reported in Table 2. Only one-quarter of adolescents reported consistent access to strong parental supervision, and less than one in five received consistent positive parenting. A third of adolescents reported living in abuse-free homes at both baseline and follow-up, while nearly three-quarters accessed government cash transfers consistently at baseline and follow-up. Only 2.1% of the sample had correct HIV knowledge, which may be explained due to the young age of the participants.

### Provisions Associated with Exposure to Sexual Risk

Four provisions were associated with reduced odds of exposure to sexual risk. Adolescents receiving consistent parenting/caregiver supervision were 47% less likely to report sexual risk exposure (OR 0.53 95%CI 0.35–0.80 p = 0.002). Living in abuse-free homes at both baseline and follow-up was associated with a 45% reduction in the odds of sexual risk exposure (OR 0.55 95%CI 0.37–0.81 p = 0.002). Receiving at least one meal a day at school consistently reduced the odds of sexual risk exposure by 45% (OR 0.55 95%CI 0.35–0.88 p = 0.012). HIV prevention knowledge was also associated with reduced sexual risk exposure, with a stronger impact on consistent correct HIV prevention knowledge: adolescents who had the correct knowledge at baseline and follow-up were 57% less likely to report sexual risk exposure compared to no access (OR 0.43 95%CI 0.21–0.88 p = 0.021), while those who had inconsistent HIV prevention knowledge were likely to report a 32% reduction in sexual risk exposure (OR 0.68 95%CI 0.47–0.99 p = 0.044).

Combinations of consistent provisions, compared with single consistent provisions, were associated with lower odds of sexual risk exposure (Supplementary Table 1). Consistent access to each factor reduced the probability of reporting sexual risk exposure from 12.9% (95%CI 7.2–18.7) without any access to provisions, to 1.0% (95%CI 0.2–1.8) with consistent access to all four provisions (Fig. 1). Most of the participants accessed at least one provision (51.9%), followed by 30.8% of adolescents that accessed two provisions, 8.5% who accessed three of the four provisions and a handful (n = 7, 0.2%) who accessed all four of the provisions. About 10% of the adolescents did not access any of the provisions.

**Fig. 2:**
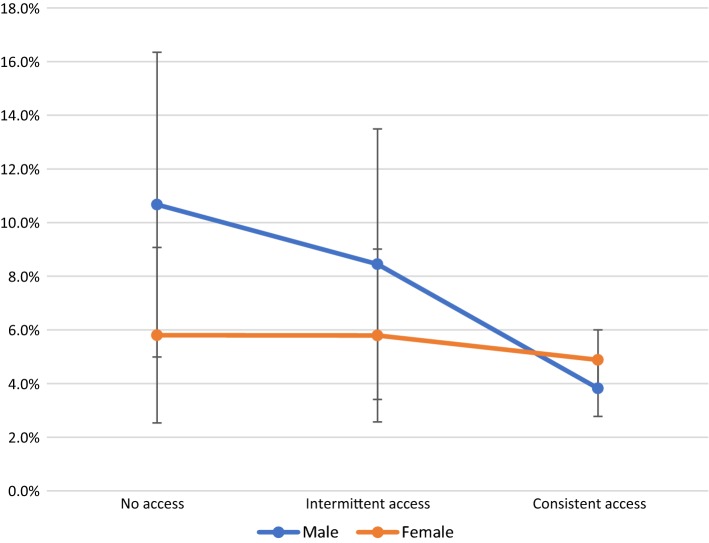
Probability of incident sexual risk exposure by gender and access to school feeding scheme

Older age and living in the Eastern Cape province remained significantly associated with exposure to sexual risk in the final model. There were no two- or three-way interactions among the four provisions significantly associated to reduced exposure to sexual risk (data not shown). Gender moderated the effect of the association between school feeding and sexual risk exposure, with no other significant moderation effects (Supplementary Tables 2 and 3). The effect of accessing school feeding (whether intermittently or consistently) was stronger among young adolescent boys than adolescent girls, with consistent access to school feeding among boys resulting in the highest reduction in the probability of reporting exposure to sexual risk (Fig. 2).

## Discussion

The findings from this secondary analysis of data from two large, longitudinal studies in South Africa demonstrate significant associations between four provisions and reduced exposure to sexual risk in early adolescence. These longitudinal data provide additional evidence that can shape current and future programming for HIV prevention for adolescents in South Africa and similar HIV risk contexts. It capitalizes on two large-scale prospective surveys of early adolescents to draw lessons on factors that shape the onset of exposure to sexual risk and HIV, in the transition from early to middle adolescence, which are critical for programming for this early age group [50]. Most adolescent-focused research and programming targets older adolescents, particularly 15- to 24-year-old girls and young women, but these new findings suggest an additional impact of providing such programs to younger girls and boys. These provisions—parental supervision, abuse-free homes, school feeding and HIV prevention knowledge—could be provided through home- and school-based programming.

Four provisions were associated with reduced odds of sexual risk exposure. Each provision individually reduced the odds of sexual risk exposure from 32% (OR 0.68 95%CI 0.47–0.99 p = 0.044) to 57% (OR 0.43 95%CI 0.21–0.88 p = 0.021), highlighting the importance of accessing provisions in early adolescence consistently to address long-term vulnerabilities and risks faced by South African adolescents and young people. Importantly, combinations of interventions resulted in a 92% reduction in the probability of reporting sexual risk exposure, from 12.9% (95%CI 7.2–18.7) to 1.0% (95%CI 0.2–1.8). Only two out of five participants accessed multiple provisions, although just over half accessed at least one of them. One in 12 adolescents in this sample reported sexual risk exposure over a one-year period—a high rate considering that 99% of participants were under 18 years old at baseline. Access to government cash transfers was not associated with sexual risk exposure, but this may be linked to lower access to cash transfers in the highest-risk group of older girls. Our findings confirm the importance of investing in early adolescence to reduce poorer health and educational outcomes in later adolescence [36]. This is particularly relevant considering findings from the Violence Against Children Survey suggest high rates of coercion and violence during first sexual encounters in Southern Africa [51].

An important finding of our analyses is that intermittent access to provisions—access at either baseline or follow-up, but not both—does not reduce the likelihood of sexual risk exposure, with the exception of HIV knowledge. However, *consistent* access over a full year in early adolescence to provisions may have substantial impacts in delaying sexual risk exposure. Gender did not moderate the association between the provisions and sexual risk exposure, with the exception of school feeding. This finding suggests that in early adolescence, the same combination of interventions may support both boys and girls, whether they live in rural or urban communities, to prevent sexual risk exposure and the possibility of HIV infection. The significantly stronger effect of school feeding schemes on reducing sexual risk exposure among adolescent boys needs to be further investigated. As programming increasingly focuses on 15- to 24-year-old adolescent girls, it is critical to provide consistent access to provisions that, in early adolescence, may have long-term benefits for both adolescent boys and girls.

This study has several limitations. Our sample of adolescents was still young, and consequently their reported exposure to sexual risk is lower than the risk they will experience throughout the course of adolescence and early adulthood. We can expect higher rates of sexual risk exposure among older adolescents. However, there is insufficient research with young adolescents, and this study provides critical information about this age group. Another limitation is linked to the number of provisions measured. The pooled data only included shared measures for eight possible factors. Third, this study did not collect biomarker data, such as HIV incidence using clinical test results. Nonetheless, in light of strong linkages documenting sexual risk exposure in early adolescence and HIV incidence [13, 52, 53], our findings contribute to an understanding of how to break pathways leading to HIV infection among adolescents and young people.

Despite these limitations, our longitudinal analyses with an under-researched age group provide valuable information for future programming seeking to reduce HIV incidence among adolescents and young people. While sustaining programming that reaches the highest-risk groups, namely 15- to 24-year-old young women, is essential, these findings show a substantive impact on younger adolescents—including boys—and those who have not yet initiated sexual activity or been exposed to sexual risk. They suggest that early, consistent access to protective programming at home and school—programming that focuses on reducing food insecurity, boosting caregiver-adolescent engagement, improving correct HIV prevention knowledge, and preventing violence in the home—is critical to preventing sexual risk exposure among vulnerable young people.

## Electronic supplementary material

Below is the link to the electronic supplementary material.

Supplementary material 1 (DOCX 27 kb)
