# Supplementary material for: Consistent Provisions Mitigate Exposure to Sexual Risk and HIV Among Young Adolescents in South Africa
Source: AIDS Behav. 2019 Nov 20;24(3):903–13. doi: 10.1007/s10461-019-02735-x (PMC7018679; doi:10.1007/s10461-019-02735-x)
Supplement: Supplementary file 1 — Supplementary material 1 (DOCX 27 kb) [file 10461_2019_2735_MOESM1_ESM.docx]

Supplementary tables

***Supplementary table 1 - Marginal effects***

| **Combinations of supportive/ protective provisions** | **Percentage probability** | **95% CI** |
| --- | --- | --- |
| No provision | 12.9 | 7.2-18.7 |
| School feeding only (intermittent) | 11.3 | 6.0-16.6 |
| School feeding only (consistent) | 7.6 | 5.2-10.0 |
| Parental/caregiver supervision only (intermittent) | 10.1 | 5.5-14.8 |
| Parental/caregiver supervision and school feeding (intermittent) | 8.8 | 4.6-13.0 |
| Parental/caregiver supervision (intermittent) and school meals (consistent) | 5.9 | 4.0-7.8 |
| Parental/caregiver supervision only (consistent) | 7.3 | 3.3-11.3 |
| Parental/caregiver supervision (consistent) and school feeding (intermittent) | 6.3 | 2.8-9.9 |
| Parental/caregiver supervision (consistent) and school feeding (consistent) | 4.2 | 2.4-5.9 |
| Abuse-free home (intermittent) | 12.1 | 6.8-17.3 |
| Abuse-free home (intermittent) and school feeding (intermittent) | 10.5 | 5.8-15.3 |
| Abuse-free home (intermittent) and school feeding (consistent) | 7.0 | 5.0-9.1 |
| Abuse-free home (intermittent) and parental/caregiver supervision (intermittent) | 9.4 | 5.3-13.5 |
| Abuse-free home (intermittent), parental/caregiver supervision (intermittent) and school feeding (intermittent) | 8.2 | 4.6-11.9 |
| Abuse-free home (intermittent), parental/caregiver supervision (intermittent) and school feeding (consistent) | 5.4 | 3.9-6.9 |
| Abuse-free home (intermittent), parental/caregiver supervision (consistent) | 6.8 | 3.3-10.2 |
| Abuse-free home (intermittent), parental/caregiver supervision (consistent) and school feeding (intermittent) | 5.9 | 2.8-8.9 |
| Abuse-free home (intermittent), parental/caregiver supervision (consistent) and school meals (consistent) | 3.9 | 2.4-5.3 |
| Abuse-free home only (consistent) | 7.5 | 3.8-11.2 |
| Abuse-free home (consistent) and school feeding (intermittent) | 6.6 | 3.2-9.9 |
| Abuse-free home (consistent) and school feeding (consistent) | 4.3 | 2.8-5.8 |
| Abuse-free home (consistent) and parental supervision (intermittent) | 5.8 | 3.0-8.6 |
| Abuse-free home (consistent), parental/caregiver supervision (intermittent) and school feeding (intermittent) | 5.0 | 2.6-7.5 |
| Abuse-free home (consistent), parental/caregiver supervision (intermittent) and school feeding (consistent) | 3.3 | 2.2-4.4 |
| Abuse-free home (consistent) and parental/caregiver supervision (consistent) | 4.1 | 1.9-6.4 |
| Abuse-free home (consistent), parental/caregiver supervision (consistent) and school feeding (intermittent) | 3.6 | 1.6-5.6 |
| Abuse-free home (consistent), parental/caregiver supervision (consistent) and school feeding (consistent) | 2.3 | 1.4-3.3 |
| HIV prevention education (intermittent) | 9.2 | 4.1-14.3 |
| HIV prevention education (intermittent) and school feeding (intermittent) | 8.0 | 3.5-12.6 |
| HIV prevention education (intermittent) and school feeding (consistent) | 5.3 | 2.9-7.8 |
| HIV prevention education (intermittent) and parental/caregiver supervision (intermittent) | 7.2 | 3.2-11.2 |
| HIV prevention education (intermittent), parental/caregiver supervision (intermittent) and school feeding (intermittent) | 6.2 | 2.7-9.7 |
| HIV prevention education (intermittent), parental/caregiver supervision (intermittent) and school feeding (consistent) | 4.1 | 2.2-6.0 |
| HIV prevention education (intermittent) and parental/caregiver supervision (consistent) | 5.1 | 1.9-8.3 |
| HIV prevention education (intermittent), parental/caregiver supervision (consistent) and school feeding (intermittent) | 4.4 | 1.6-7.3 |
| HIV prevention education (intermittent), parental/caregiver supervision (consistent) and school feeding (consistent) | 2.9 | 1.4-4.4 |
| HIV prevention education (intermittent) and abuse-free home (intermittent) | 8.6 | 3.9-13.3 |
| HIV prevention education (intermittent), abuse-free home (intermittent) and school feeding (intermittent) | 7.5 | 3.3-11.6 |
| HIV prevention education (intermittent), abuse-free home (intermittent) and school feeding (consistent) | 4.9 | 2.7-7.1 |
| HIV prevention education (intermittent), abuse-free home (intermittent) and parental/caregiver supervision (intermittent) | 6.6 | 3.1-10.2 |
| HIV prevention education (intermittent), abuse-free home (intermittent), parental/caregiver supervision (intermittent) and school feeding (intermittent) | 5.8 | 2.6-8.9 |
| HIV prevention education (intermittent), abuse-free home (intermittent), parental/caregiver supervision (intermittent) and school feeding (consistent) | 3.8 | 2.2-5.4 |
| HIV prevention education (intermittent), abuse-free home (intermittent) and parental/caregiver supervision (consistent) | 4.7 | 1.9-7.6 |
| HIV prevention education (intermittent), abuse-free home (intermittent), parental/caregiver supervision (consistent) and school feeding (intermittent) | 4.1 | 1.6-6.6 |
| HIV prevention education (intermittent), abuse-free home (intermittent), parental/caregiver supervision (consistent) and school feeding (consistent) | 2.7 | 1.4-4.0 |
| HIV prevention education (intermittent) and abuse-free home (consistent) | 5.3 | 2.1-8.4 |
| HIV prevention education (intermittent), abuse-free home (consistent) and school feeding (intermittent) | 4.6 | 1.8-7.3 |
| HIV prevention education (intermittent), abuse-free home (consistent) and school feeding (consistent) | 3.0 | 1.5-4.5 |
| HIV prevention education (intermittent), abuse-free home (consistent) and parental /caregiver supervision (intermittent) | 4.1 | 1.7-6.4 |
| HIV prevention education (intermittent), abuse-free home (consistent), parental/caregiver supervision (intermittent) and school feeding (intermittent) | 3.5 | 1.5-5.5 |
| HIV prevention education (intermittent), abuse-free home (consistent), parental/caregiver supervision (intermittent) and school feeding (consistent) | 2.3 | 1.2-3.4 |
| HIV prevention education (intermittent), abuse-free home (consistent), parental/caregiver supervision (consistent) | 2.9 | 1.1-4.7 |
| HIV prevention education (intermittent), abuse-free home (consistent), parental/caregiver supervision (consistent) and school feeding (intermittent) | 2.5 | 0.9-4.1 |
| HIV prevention education (intermittent), abuse-free home (consistent), parental/caregiver supervision (consistent) and school feeding (consistent) | 1.6 | 0.8-2.4 |
| HIV prevention education only (consistent) | 6.0 | 1.1-10.9 |
| HIV prevention education (consistent) and school feeding (intermittent) | 5.2 | 0.9-9.4 |
| HIV prevention education (consistent) and school feeding (consistent) | 3.4 | 0.8-6.0 |
| HIV prevention education (consistent) and parental/caregiver supervision (intermittent) | 4.6 | 0.8-8.4 |
| HIV prevention education (consistent), parental/caregiver supervision (intermittent) and school feeding (intermittent) | 4.0 | 0.7-7.3 |
| HIV prevention education (consistent), parental/caregiver supervision (intermittent) and school feeding (consistent) | 2.6 | 0.6-4.6 |
| HIV prevention education (consistent) and parental/caregiver supervision (consistent) | 3.3 | 0.4-6.1 |
| HIV prevention education (consistent), parental/caregiver supervision (consistent) and school feeding (intermittent) | 2.8 | 0.3-5.3 |
| HIV prevention education (consistent), parental/caregiver supervision (consistent) and school feeding (consistent) | 1.8 | 0.3-3.3 |
| HIV prevention education (consistent) and abuse-free home (intermittent) | 5.6 | 1.1-10.0 |
| HIV prevention education (consistent), abuse-free home (intermittent) and school feeding (intermittent) | 4.8 | 1.0-8.6 |
| HIV prevention education (consistent), abuse-free home (intermittent) and school feeding (consistent) | 3.1 | 0.8-5.5 |
| HIV prevention education (consistent), abuse-free home (intermittent) and parental/caregiver supervision (intermittent) | 4.3 | 0.8-7.7 |
| HIV prevention education (consistent), abuse-free home (intermittent), parental/caregiver supervision (intermittent) and school feeding (intermittent) | 3.7 | 0.7-6.7 |
| HIV prevention education (consistent), abuse-free home (intermittent), parental/caregiver supervision (intermittent) and school feeding (consistent) | 2.4 | 0.6-4.2 |
| HIV prevention education (consistent), abuse-free home (intermittent) and parental/caregiver supervision (consistent) | 3.0 | 0.4-5.6 |
| HIV prevention education (consistent), abuse-free home (intermittent), parental/caregiver supervision (consistent) and school feeding (intermittent) | 2.6 | 0.4-4.8 |
| HIV prevention education (consistent), abuse-free home (intermittent), parental/caregiver supervision (consistent) and school feeding (consistent) | 1.7 | 0.4-3.0 |
| HIV prevention education (consistent) and abuse-free home (consistent) | 3.4 | 0.5-6.3 |
| HIV prevention education (consistent), abuse-free home (consistent) and school feeding (intermittent) | 2.9 | 0.4-5.4 |
| HIV prevention education (consistent), abuse-free home (consistent) and school feeding (consistent) | 1.9 | 0.4-3.4 |
| HIV prevention education (consistent), abuse-free home (consistent) and parental/caregiver supervision (intermittent) | 2.6 | 0.4-4.8 |
| HIV prevention education (consistent), abuse-free home (consistent), parental/caregiver supervision (intermittent) and school feeding (intermittent) | 2.2 | 0.3-4.1 |
| HIV prevention education (consistent), abuse-free home (consistent), parental/caregiver supervision (intermittent) and school feeding (consistent) | 1.4 | 0.3-2.6 |
| HIV prevention education (consistent), abuse-free home (consistent), parental/caregiver supervision (consistent) | 1.8 | 0.2-3.4 |
| HIV prevention education (consistent), abuse-free home (consistent), parental/caregiver supervision (consistent) and school feeding (intermittent) | 1.6 | 0.2-3.0 |
| HIV prevention education (consistent), abuse-free home (consistent), parental/caregiver supervision (consistent) and school feeding (consistent) | 1.0 | 0.2-1.8 |

***Supplementary table 2 – Final model with moderation effects of gender***

|  | **Odds ratio** | **p-value** | **95% CI** |
| --- | --- | --- | --- |
| Age | 1.43 | <0.0001 | 1.33-1.53 |
| *Gender (female)* | 0.47 | 0.131 | 0.18-1.25 |
| Province (reference category: Eastern Cape) | | | |
| *Western Cape* | 0.16 | <0.0001 | 0.11-0.24 |
| *Mpumalanga* | 0.07 | <0.0001 | 0.05-0.11 |
| Parental/caregiver support - Strong parental supervision^1^ | | | |
| *Intermittent access* | 0.71 | 0.146 | 0.45-1.12 |
| *Consistent access* | 0.41 | 0.007 | 0.22-0.79 |
| Violence prevention | | | |
| *Intermittent access* | 0.91 | 0.722 | 0.54-1.54 |
| *Consistent access* | 0.54 | 0.035 | 0.31-0.96 |
| Educational subsidies – School meals | | | |
| *Intermittent access* | 0.77 | 0.561 | 0.32-1.84 |
| *Consistent access* | 0.33 | 0.001 | 0.17-0.64 |
| HIV prevention education | | | |
| *Intermittent access* | 0.93 | 0.777 | 0.54-1.58 |
| *Consistent access* | 0.37 | 0.147 | 0.10-1.41 |
| Parental/caregiver support - Strong parental supervision * Gender | | | |
| *Intermittent access, female* | 1.12 | 0.716 | 0.61-2.06 |
| *Consistent access, female* | 1.53 | 0.306 | 0.68-3.45 |
| Violence prevention * Gender | | | |
| *Intermittent access, female* | 1.02 | 0.956 | 0.51-2.04 |
| *Consistent access, female* | 1.01 | 0.978 | 0.47-2.16 |
| Educational subsidies – School meals * Gender | | | |
| *Intermittent access, female* | 1.29 | 0.674 | 0.39-4.22 |
| *Consistent access, female* | 2.50 | 0.040 | 1.04-6.00 |
| HIV prevention education * Gender | | | |
| *Intermittent access, female* | 0.57 | 0.106 | 0.28-1.13 |
| *Consistent access, female* | 0.17 | 0.841 | 0.25-5.56 |

^1^ All results for provisions accessed intermittently or consistently use no access as a reference category.

***Supplementary table 3 – Final model with moderation effects of rural residence***

| **Outcome measure: incident high-risk sex** | **Odds ratio (OR)** | **p-value** | **95% CI** |
| --- | --- | --- | --- |
| Age | 1.42 | <0.0001 | 1.32-1.52 |
| Rural residence | 2.39 | 0.168 | 0.69-8.22 |
| Province (reference category: Eastern Cape) | | | |
| *Western Cape* | 0.18 | <0.0001 | 0.13-0.27 |
| *Mpumalanga* | 0.08 | <0.0001 | 0.05-0.13 |
| Parental/caregiver support - Strong parental supervision^1^ | | | |
| *Intermittent access* | 0.80 | 0.243 | 0.55-1.16 |
| *Consistent access* | 0.48 | 0.003 | 0.29-0.78 |
| Violence prevention | | | |
| *Intermittent access* | 0.99 | 0.965 | 0.65-1.51 |
| *Consistent access* | 0.63 | 0.047 | 0.40-0.99 |
| Educational subsidies – School feeding | | | |
| *Intermittent access* | 1.01 | 0.967 | 0.51-2.01 |
| *Consistent access* | 0.71 | 0.197 | 0.42-1.20 |
| HIV prevention education | | | |
| *Intermittent access* | 0.66 | 0.047 | 0.43-0.99 |
| *Consistent access* | 0.41 | 0.035 | 0.18-0.94 |
| Parental/caregiver support - Strong parental supervision * Residential location | | | |
| *Intermittent access, rural* | 0.85 | 0.613 | 0.44-1.62 |
| *Consistent access, rural* | 1.48 | 0.363 | 0.64-3.43 |
| Violence prevention * Residential location | | | |
| *Intermittent access, rural* | 0.81 | 0.576 | 0.40-1.67 |
| *Consistent access, rural* | 0.66 | 0.317 | 0.29-1.50 |
| Educational subsidies – School feeding * Residential location | | | |
| *Intermittent access, rural* | 0.44 | 0.272 | 0.10-1.90 |
| *Consistent access, rural* | 0.37 | 0.096 | 0.11-1.19 |
| HIV prevention education * Residential location | | | |
| *Intermittent access, rural* | 1.23 | 0.611 | 0.56-2.71 |
| *Consistent access, rural* | 1.43 | 0.664 | 0.28-7.22 |

^1^ All results for provisions accessed intermittently or consistently use no access as a reference category.
